# 5-Chloro-1-phenyl-1*H*-pyrazol-4-amine

**DOI:** 10.1107/S1600536811032065

**Published:** 2011-08-11

**Authors:** Artur Korzański, Pawel Wagner, Maciej Kubicki

**Affiliations:** a Department of Chemistry, Adam Mickiewicz University, Grunwaldzka 6, 60–780 Poznań, Poland; b The ARC Centre of Excellence for Electromaterials Science, Intelligent Polymer Research Institute, University of Wollongong, Innovation Campus, Squires Way, Fairy Meadow, NSW 2519, Australia

## Abstract

In the crystal structure of the title compound, C_9_H_8_ClN_3_, amino–pyrazole N—H⋯N hydrogen bonds connect the mol­ecules along the [010] direction; the chains interact with each other only by van der Waals-type inter­actions. The pyrazole and phenyl rings are inclined at a dihedral angle of 45.65 (6)°

## Related literature

For the synthesis, see: Tallec *et al.* (2000 ▶). For other 4-amino­pyrazoles, see: Infantes *et al.* (1998 ▶, 1999 ▶); Schmidt *et al.* (2001 ▶). For a description of the Cambridge Structural Database, see: Allen (2002 ▶).
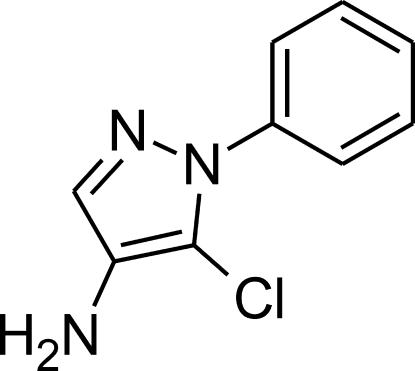


## Experimental

### 

#### Crystal data

C_9_H_8_ClN_3_
*M* *_r_* = 193.63
Monoclinic, 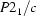

*a* = 3.8926 (6) Å
*b* = 9.9679 (13) Å
*c* = 22.617 (2) Å
β = 92.795 (11)°
*V* = 876.52 (19) Å^3^
*Z* = 4
Mo *K*α radiation
μ = 0.39 mm^−1^
*T* = 295 K
0.4 × 0.07 × 0.06 mm

#### Data collection

Agilent Xcalibur Sapphire2 diffractometer
Absorption correction: multi-scan (*CrysAlis PRO*; Agilent, 2010 ▶) *T*_min_ = 0.853, *T*_max_ = 1.000
5036 measured reflections
1879 independent reflections
1340 reflections with *I* > 2σ(*I*)
*R*_int_ = 0.035

#### Refinement

*R*[*F* ^2^ > 2σ(*F* ^2^)] = 0.040
*wR*(*F* ^2^) = 0.091
*S* = 1.02
1879 reflections
132 parameters
H atoms treated by a mixture of independent and constrained refinement
Δρ_max_ = 0.17 e Å^−3^
Δρ_min_ = −0.21 e Å^−3^

### 

Data collection: *CrysAlis PRO* (Agilent, 2010 ▶); cell refinement: *CrysAlis PRO*; data reduction: *CrysAlis PRO*; program(s) used to solve structure: *SIR92* (Altomare *et al.*, 1993 ▶); program(s) used to refine structure: *SHELXL97* (Sheldrick, 2008 ▶); molecular graphics: *SHELXTL* (Sheldrick, 2008 ▶) and *Mercury* (Macrae *et al.*, 2008 ▶); software used to prepare material for publication: *SHELXL97*.

## Supplementary Material

Crystal structure: contains datablock(s) I, global. DOI: 10.1107/S1600536811032065/rk2288sup1.cif

Structure factors: contains datablock(s) I. DOI: 10.1107/S1600536811032065/rk2288Isup2.hkl

Supplementary material file. DOI: 10.1107/S1600536811032065/rk2288Isup3.cml

Additional supplementary materials:  crystallographic information; 3D view; checkCIF report

## Figures and Tables

**Table 1:** Hydrogen-bond geometry (Å, °)

*D*—H⋯*A*	*D*—H	H⋯*A*	*D*⋯*A*	*D*—H⋯*A*
N4—H41⋯N2^i^	0.85 (3)	2.31 (3)	3.144 (3)	169 (3)

Symmetry code: (i) 
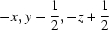
.

**Fig. 1.**
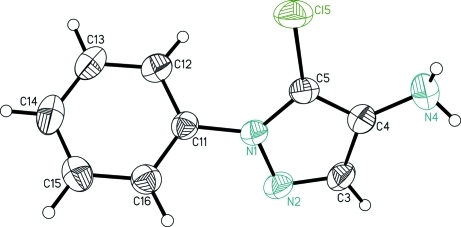
The molecular structure of title compound with the atom numbering scheme. The displacement ellipsoids are drawn at 50% probability level. Hydrogen atoms are depicted as spheres of arbitrary radius.

**Fig. 2.**
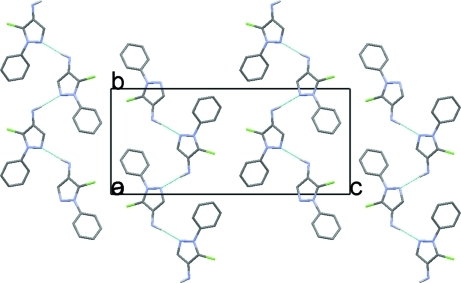
The crystal packing as seen along [1 0 0] direction; hydrogen bonds are shown as dashed lines.

## supplementary crystallographic information

### Comment

In the course of our studies on small heterocyclic ring derivatives we have determined the crystal structure of another member of 1–phenyl–4–amino–5–chloro–pyrazole, I, (Scheme 1). Some crystal structures of 4–aminopyrazoles were also reported, *e.g.* two polymorphic forms of 4–aminopyrazole (Infantes *et al.*, 1998), 3,5–dimethyl–4–aminopyrazole (Infantes *et al.*, 1999), and 4–amino–3,5–dinitropyrazole and it s dimethylsulfoxide solvate (Schmidt *et al.*, 2001).

The Fig. 1 shows the perspective view of I. Two planar fragments, pyrazole (maximum deviation 0.0025 (12)Å) and phenyl (0.0082 (13)Å) rings are inclined by 45.65 (6)°. This is quite a typical value, for 241 compounds with similar structural fragment (5–substituted pyrazole, non–*o*–substituted phenyl) found in the Cambridge Structural Database (Allen, 2002; ver. 5.32 of Nov. 2010, last update May 2011) mean value of the twist angle is around 43°, and such is also the median value. The NH_2_–group is quite significantly twisted with respect to the pyrazole ring plane, the dihedral angle between two planes is 48 (2)°.

In the crystal structure the relatively weak N4—H41···N2^i^ hydrogen bonds join 2_1_ screw–related molecules into the C(5) chains along *y*–direction. Symmetry code: (i) -*x*, *y*-1/2, -*z*+1/2. There are no other specific interactions, so apparently the chains are organized into three–dimensional structure by van der Waals forces.

### Experimental

The compound was synthesized by electrochemical reduction of 4–nitro–1–phenylpyrazole in diluted hydrochloric acid to corresponding hydroxylamine and its *in situ* nucleophilic transformation into 5–chloro derivative. The compound was separated from post–reaction mixture with low yield. (Tallec *et al.*, 2000).

### Refinement

Hydrogen atoms from NH_2_–group were found in the difference Fourier maps and freely refined with isotropic displacement parameters. All other hydrogen atoms were placed in idealized positions (C—H distance 0.93Å) and refined as a riding model with their *U*_iso_ = 1.2*U*_eq_(C).

### Crystal data

**Table:**

C_9_H_8_ClN_3_	*F*(000) = 400
*M**_r_* = 193.63	*D*_x_ = 1.467 Mg m^−^^3^
Monoclinic, *P*2_1_/*c*	Mo *K*α radiation, λ = 0.71073 Å
Hall symbol: -P 2ybc	Cell parameters from 1285 reflections
*a* = 3.8926 (6) Å	θ = 2.0–27.8°
*b* = 9.9679 (13) Å	µ = 0.39 mm^−^^1^
*c* = 22.617 (2) Å	*T* = 295 K
β = 92.795 (11)°	Needle, colourless
*V* = 876.52 (19) Å^3^	0.4 × 0.07 × 0.06 mm
*Z* = 4	

### Data collection

**Table:**

Agilent Xcalibur Sapphire2 diffractometer	1879 independent reflections
Radiation source: Enhance (Mo) X–ray Source	1340 reflections with *I* > 2σ(*I*)
graphite	*R*_int_ = 0.035
Detector resolution: 8.1929 pixels mm^-1^	θ_max_ = 27.0°, θ_min_ = 3.6°
ω scans	*h* = −4→4
Absorption correction: multi-scan (*CrysAlis PRO*; Agilent, 2010)	*k* = −12→12
*T*_min_ = 0.853, *T*_max_ = 1.000	*l* = −28→28
5036 measured reflections	

### Refinement

**Table:**

Refinement on *F*^2^	Primary atom site location: structure-invariant direct methods
Least-squares matrix: full	Secondary atom site location: difference Fourier map
*R*[*F*^2^ > 2σ(*F*^2^)] = 0.040	Hydrogen site location: inferred from neighbouring sites
*wR*(*F*^2^) = 0.091	H atoms treated by a mixture of independent and constrained refinement
*S* = 1.02	*w* = 1/[σ^2^(*F*_o_^2^) + (0.0382*P*)^2^ + 0.093*P*] where *P* = (*F*_o_^2^ + 2*F*_c_^2^)/3
1879 reflections	(Δ/σ)_max_ < 0.001
132 parameters	Δρ_max_ = 0.17 e Å^−^^3^
0 restraints	Δρ_min_ = −0.21 e Å^−^^3^

### Special details

**Table:**

Geometry. All s.u.'s (except the s.u. in the dihedral angle between two l.s. planes) are estimated using the full covariance matrix. The cell s.u.'s are taken into account individually in the estimation of s.u.'s in distances, angles and torsion angles; correlations between s.u.'s in cell parameters are only used when they are defined by crystal symmetry. An approximate (isotropic) treatment of cell s.u.'s is used for estimating s.u.'s involving l.s. planes.
Refinement. Refinement of *F*^2^ against ALL reflections. The weighted *R*–factor *wR* and goodness of fit *S* are based on *F*^2^, conventional *R*–factors *R* are based on *F*, with *F* set to zero for negative *F*^2^. The threshold expression of *F*^2^ > 2σ(*F*^2^) is used only for calculating *R*–factors(gt) *etc*. and is not relevant to the choice of reflections for refinement. *R*–factors based on *F*^2^ are statistically about twice as large as those based on *F*, and *R*–factors based on ALL data will be even larger.

### Fractional atomic coordinates and isotropic or equivalent isotropic displacement parameters (Å^2^)

**Table:**

	*x*	*y*	*z*	*U*_iso_*/*U*_eq_	
N1	0.2024 (4)	1.05610 (15)	0.15336 (6)	0.0333 (4)	
C11	0.2744 (4)	1.17917 (18)	0.12513 (8)	0.0323 (4)	
C12	0.1587 (5)	1.2018 (2)	0.06707 (8)	0.0398 (5)	
H12	0.0415	1.1350	0.0457	0.043 (6)*	
C13	0.2187 (5)	1.3236 (2)	0.04152 (9)	0.0476 (5)	
H13	0.1442	1.3391	0.0024	0.070 (7)*	
C14	0.3879 (5)	1.4228 (2)	0.07319 (10)	0.0497 (6)	
H14	0.4254	1.5056	0.0558	0.060 (7)*	
C15	0.5023 (5)	1.3995 (2)	0.13093 (9)	0.0457 (5)	
H15	0.6165	1.4670	0.1524	0.046 (6)*	
C16	0.4486 (5)	1.27721 (19)	0.15698 (8)	0.0377 (4)	
H16	0.5292	1.2610	0.1957	0.039 (5)*	
N2	0.0761 (4)	1.05812 (17)	0.20821 (6)	0.0400 (4)	
C3	0.0204 (5)	0.9308 (2)	0.22098 (8)	0.0414 (5)	
H3	−0.0687	0.9022	0.2563	0.057 (6)*	
C4	0.1090 (5)	0.8444 (2)	0.17613 (8)	0.0400 (5)	
N4	0.0706 (7)	0.7062 (2)	0.17291 (11)	0.0673 (7)	
H42	0.231 (7)	0.668 (3)	0.1551 (14)	0.096 (12)*	
H41	0.059 (7)	0.670 (3)	0.2067 (13)	0.090 (10)*	
C5	0.2237 (5)	0.92795 (19)	0.13352 (7)	0.0343 (4)	
Cl5	0.39097 (13)	0.88538 (6)	0.06812 (2)	0.04946 (19)	

### Atomic displacement parameters (Å^2^)

**Table:**

	*U*^11^	*U*^22^	*U*^33^	*U*^12^	*U*^13^	*U*^23^
N1	0.0408 (8)	0.0330 (9)	0.0265 (7)	0.0010 (7)	0.0052 (7)	−0.0009 (7)
C11	0.0326 (9)	0.0331 (10)	0.0316 (9)	0.0015 (8)	0.0051 (8)	0.0015 (8)
C12	0.0402 (10)	0.0455 (12)	0.0339 (10)	0.0005 (10)	0.0034 (8)	0.0001 (9)
C13	0.0519 (12)	0.0535 (14)	0.0378 (11)	0.0064 (11)	0.0082 (10)	0.0132 (10)
C14	0.0514 (12)	0.0404 (13)	0.0588 (14)	0.0034 (10)	0.0186 (11)	0.0133 (11)
C15	0.0459 (11)	0.0366 (12)	0.0552 (13)	−0.0017 (10)	0.0071 (10)	−0.0038 (10)
C16	0.0392 (10)	0.0368 (11)	0.0370 (10)	0.0032 (9)	0.0016 (8)	−0.0018 (9)
N2	0.0506 (9)	0.0423 (10)	0.0278 (8)	0.0028 (8)	0.0086 (7)	0.0006 (7)
C3	0.0526 (12)	0.0422 (12)	0.0300 (9)	0.0003 (10)	0.0078 (9)	0.0072 (9)
C4	0.0479 (11)	0.0361 (11)	0.0356 (10)	0.0021 (9)	−0.0021 (9)	0.0025 (9)
N4	0.113 (2)	0.0338 (11)	0.0560 (14)	−0.0017 (12)	0.0120 (14)	0.0028 (10)
C5	0.0367 (10)	0.0377 (11)	0.0283 (9)	0.0018 (8)	0.0007 (8)	−0.0021 (8)
Cl5	0.0591 (3)	0.0560 (4)	0.0338 (3)	0.0070 (3)	0.0076 (2)	−0.0094 (2)

### Geometric parameters (Å, °)

**Table:**

N1—N2	1.3566 (19)	C15—C16	1.375 (3)
N1—C5	1.358 (2)	C15—H15	0.9300
N1—C11	1.417 (2)	C16—H16	0.9300
C11—C16	1.373 (2)	N2—C3	1.322 (2)
C11—C12	1.386 (2)	C3—C4	1.387 (3)
C12—C13	1.369 (3)	C3—H3	0.9300
C12—H12	0.9300	C4—C5	1.365 (3)
C13—C14	1.371 (3)	C4—N4	1.387 (3)
C13—H13	0.9300	N4—H42	0.85 (3)
C14—C15	1.379 (3)	N4—H41	0.85 (3)
C14—H14	0.9300	C5—Cl5	1.6988 (18)
			
N2—N1—C5	110.30 (15)	C14—C15—H15	119.8
N2—N1—C11	119.19 (15)	C11—C16—C15	119.21 (18)
C5—N1—C11	130.45 (15)	C11—C16—H16	120.4
C16—C11—C12	120.69 (17)	C15—C16—H16	120.4
C16—C11—N1	118.89 (15)	C3—N2—N1	104.85 (15)
C12—C11—N1	120.37 (17)	N2—C3—C4	112.76 (17)
C13—C12—C11	119.35 (19)	N2—C3—H3	123.6
C13—C12—H12	120.3	C4—C3—H3	123.6
C11—C12—H12	120.3	C5—C4—C3	103.84 (18)
C12—C13—C14	120.42 (19)	C5—C4—N4	127.4 (2)
C12—C13—H13	119.8	C3—C4—N4	128.7 (2)
C14—C13—H13	119.8	C4—N4—H42	113 (2)
C13—C14—C15	119.9 (2)	C4—N4—H41	113 (2)
C13—C14—H14	120.0	H42—N4—H41	108 (3)
C15—C14—H14	120.0	N1—C5—C4	108.25 (16)
C16—C15—C14	120.4 (2)	N1—C5—Cl5	123.71 (14)
C16—C15—H15	119.8	C4—C5—Cl5	127.94 (16)
			
N2—N1—C11—C16	45.7 (2)	C11—N1—N2—C3	176.97 (15)
C5—N1—C11—C16	−137.71 (19)	N1—N2—C3—C4	0.4 (2)
N2—N1—C11—C12	−131.79 (18)	N2—C3—C4—C5	−0.4 (2)
C5—N1—C11—C12	44.8 (3)	N2—C3—C4—N4	−176.6 (2)
C16—C11—C12—C13	−0.2 (3)	N2—N1—C5—C4	0.1 (2)
N1—C11—C12—C13	177.24 (16)	C11—N1—C5—C4	−176.81 (17)
C11—C12—C13—C14	−0.8 (3)	N2—N1—C5—Cl5	−176.49 (12)
C12—C13—C14—C15	0.8 (3)	C11—N1—C5—Cl5	6.7 (3)
C13—C14—C15—C16	0.2 (3)	C3—C4—C5—N1	0.2 (2)
C12—C11—C16—C15	1.1 (3)	N4—C4—C5—N1	176.4 (2)
N1—C11—C16—C15	−176.34 (17)	C3—C4—C5—Cl5	176.55 (14)
C14—C15—C16—C11	−1.1 (3)	N4—C4—C5—Cl5	−7.2 (3)
C5—N1—N2—C3	−0.3 (2)		

### Hydrogen-bond geometry (Å, °)

**Table:**

*D*—H···*A*	*D*—H	H···*A*	*D*···*A*	*D*—H···*A*
N4—H41···N2^i^	0.85 (3)	2.31 (3)	3.144 (3)	169 (3)

Symmetry codes: (i) −*x*, *y*−1/2, −*z*+1/2.
